# Case Report: A case of complete response to entrectinib in *NTRK* fusion gene-positive parotid gland cancer

**DOI:** 10.3389/fonc.2023.1247435

**Published:** 2023-08-04

**Authors:** Etsuko Moriyama, Sachiko Nagasu, Toshimitsu Tanaka, Yasutaka Shimotsuura, Takeharu Ono, Hirohito Umeno, Jun Akiba, Akihiko Kawahara, Fumihiko Fujita, Takumi Kawaguchi, Keisuke Miwa

**Affiliations:** ^1^ Multidisciplinary Treatment Cancer Center, Kurume University Hospital, Kurume, Japan; ^2^ Division of Gastroenterology, Department of Medicine, Kurume University School of Medicine, Kurume, Japan; ^3^ Department of Surgery, Kurume University School of Medicine, Kurume, Japan; ^4^ Department of Otolaryngology, Head and Neck Surgery, Kurume University School of Medicine, Kurume, Japan; ^5^ Department of Diagnostic Pathology, Kurume University Hospital, Kurume, Japan

**Keywords:** *NTRK* fusion gene, *ETV6-NTRK3* fusion gene, secretory carcinoma, salivary gland carcinoma, parotid gland cancer, entrectinib, next-generation sequencing, case report

## Abstract

**Introduction:**

Expression of the *NTRK* gene is rare in solid tumors but is highly prevalent in salivary gland secretory carcinomas. Here, we report a case of a complete response to entrectinib in a patient with *NTRK* fusion gene-positive parotid carcinoma.

**Case description:**

The patient was a 44-year-old man who underwent total left parotidectomy and left cervical lymph node dissection for a left parotid tumor at 24 years of age. The histopathological diagnosis was mammary analog secretory carcinoma. Postoperatively, the patient received only radiation therapy. Sixteen years after the surgery, the patient became aware of a mass in the left parotid region. A close examination revealed local recurrence and multiple cervical lymph node metastases. S-1 monotherapy was started as chemotherapy but was discontinued 3 years later because of disease progression. As there was no standard treatment, a comprehensive genomic profiling test using a next-generation sequencer was performed, and the *ETV6-NTRK3* fusion gene was identified. Entrectinib, an *NTRK* inhibitor, was immediately administered at a dose of 600 mg/day. The local recurrence rapidly shrank grossly from the beginning of treatment, and a complete response was observed 6 months later. However, creatinine levels exhibited an increase at week 68 of treatment; consequently, entrectinib dosage was lowered to 400 mg/day, leading to an immediate improvement in creatinine levels. Entrectinib was associated with additional side effects, including dysgeusia, fatigue, dizziness, and weight gain, all of which were also alleviated by the reduction in entrectinib dose. Thirty months after treatment initiation, the patient maintained a complete response and continued to receive entrectinib.

**Conclusion:**

The *NTRK* fusion gene should always be checked in the presence of salivary gland secretory carcinoma.

## 1 Introduction

In 2010, Skalova et al. reported a histological type of salivary gland carcinoma that resembled the secretory carcinoma (SC) of the mammary gland, known as mammary analog secretory carcinoma (MASC) (1). Recently, MASC was established as a new histologic type in the World Health Organization Classification of Head and Neck Tumors (4th edition) as SC (2). SCs are low-grade salivary gland tumors that grow relatively slowly (1, 3, 4); however, there is currently no effective drug therapy. Recently, entrectinib, an inhibitor of tropomyosin receptor kinase (TRKs), has been reported to be effective in treating solid tumors with *NTRK* fusion genes (5). The *ETV6-NTRK3* fusion gene has been identified in several SCs (1, 6, 7). Additionally, in Japan, a comprehensive genomic profiling (CGP) test using next-generation sequencing for solid tumors for which there is no standard treatment was covered by insurance in June 2019.

Herein, we report a case of a complete response to entrectinib in a patient with *NTRK* fusion gene-positive parotid carcinoma.

## 2 Case description

A 44-year-old man, in January 20XX (24 years old at that time), underwent left parotid pretreatment and cervical dissection for parotid carcinoma. A histopathological diagnosis of MASC was established. Postoperatively, the patient was treated with radiation therapy, but no drug therapy was administered, and the patient was followed up. In April 20XX+16, 16 years after the initial surgery, the patient visited our otolaryngology clinic because of a palpable mass in the left parotid region. After close examination, the patient was diagnosed with local recurrence and cervical lymph node metastasis. However, complete resection of the lymph nodes proved to be challenging. Unfortunately, there were no recommend drug therapies available for effective treatment. In response to the patient’s request for oral treatment, oral S-1 therapy was initiated. However, local recurrence gradually increased and lung metastasis appeared in October 20XX+19; therefore, S-1 oral therapy was discontinued, and a biopsy of cervical lymph node metastasis was performed simultaneously.

**Figure 1:**
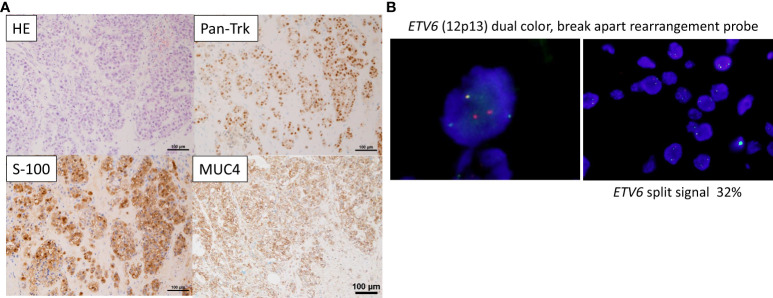
Pathological findings **(A)** On Hematoxylin & Eosin (HE) staining, tumor cells with well-defined nucleoli and a pale eosinophilic, partially vacuolated cytoplasm resemble a microcystic form, presenting a histology similar to the microcystic form of acinic cell carcinoma. Immunostaining showing that Pan-Trk and S-100, and MUC4 are widely expressed. **(B)** Fluorescence *in situ* hybridization (FISH) using the *ETV6* break apart probe examined for the presence of yellow (red/green fusion) or green/red fluorescent signals in the tumor cells. Yellow signals are negative, and separate red and green signals are positive. *ETV6* split signal of 32% is seen (cutoff, 10%).

The pathological diagnosis of the biopsied tissue was SC, which was similar to that at the time of the initial surgery. Immunostaining for S-100, MUC4, and Pan-Trk showed widely positive results (**Figure 1A**), while immunostaining for mammaglobin revealed partially positive result. Subsequently, fluorescence *in situ* hybridization (FISH) with an *ETV6* (12p13) break-apart probe (Vysis ETV6 Break Apart FISH Probe Kit, Abbott) using a previously reported method (8) revealed split signals in the nuclei (**Figure 1B**). These findings supported the diagnosis of SC. In Japan, the use of TRK inhibitors requires *ETV6-NTRK3* fusion analysis using next-generation sequencing. Finally, a CGP test using FoundationOne^®^ CDx (Foundation Medicine, Cambridge, MA, USA) was performed, and the *ETV6-NTRK3* fusion gene was identified.

**Figure 2:**
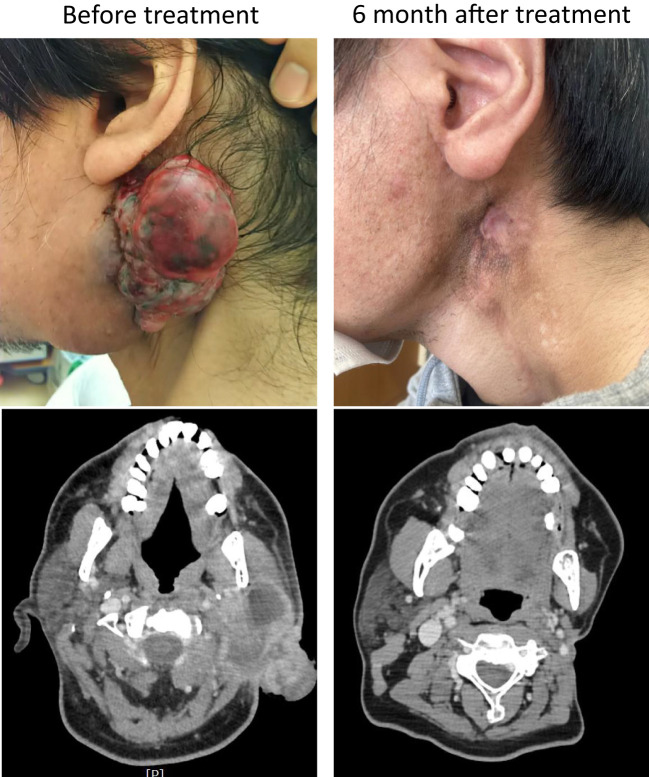
Gross and computed tomography (CT) images. The upper panels show the gross findings of the local recurrence site before and after treatment, and the lower panels show the CT images of the same site before and after treatment.

The patient was started on entrectinib therapy (600 mg/day) in January 20XX +20. The patients’ pretreatment blood test results are shown in **Supplementary Table 1**; there were no problematic findings. However, the patient had a recurrent tumor approximately 7 cm in length in the left parotid region, which had partially self-disintegrated and showed effusion; therefor, he lived with a gauze over his neck at all times. After treatment, the local recurrence in the left parotid region shrank rapidly and completely disappeared 6 months later (**Figure 2**, upper row). Computed tomography (CT) findings also showed that the local recurrent lesion had shrunk deeply after 2 months of treatment and completely disappeared after 6 months of treatment (**Figure 2**, lower row). Simultaneously, the pulmonary metastases disappeared, and the CT images revealed a complete response.

**Figure 3:**
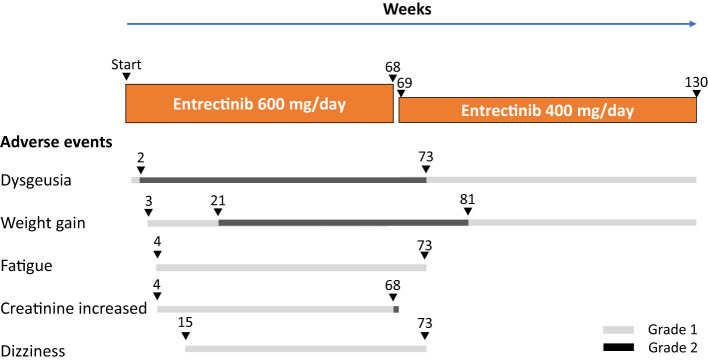
Adverse events with entrectinib and patients’ clinical course.

Adverse events experienced during entrectinib treatment and the patients’ clinical course are illustrated in **Figure 3**. Adverse events with entrectinib included grade 1 (CTCAE version 5.0) dysgeusia on day 4 of treatment, which worsened to grade 2 by week 2; however, the oral intake was good and tolerable. In addition, grade 1 weight gain was observed at week 3 of treatment, grade 1 fatigue and creatinine levels increased at week 4, grade 1 dizziness was observed at week 15, and grade 2 weight gain increased at week 21, all within acceptable limits; entrectinib was continued at a dose of 600 mg/day. However, an increase in grade 2 creatinine levels was observed at week 68 of treatment. Entrectinib was withdrawn for 1 week, and creatinine levels immediately improved to baseline values; therefore, entrectinib was reduced to 400 mg/day and continued from April 20XX+21. After the entrectinib dose reduction, all adverse events decreased, and the patient was able to go about his daily life, including work, without any problems.

Thirty months after the start of treatment, the patient is still receiving entrectinib therapy, and complete resolution of the recurrent lesions has been sustained on imaging and gross examination.

## 3 Discussion

The mean age of 91 patients with MASC was reported to be 44.7 years (range: 14–77 years), with 70% occurring in the parotid gland (9). The clinical course of salivary gland SC is characterized by a moderate risk of local recurrence, as well as a low risk of distant metastasis. Moreover, SC is generally considered a low-grade cancer with an overall good prognosis (1, 3, 4). However, some patients may develop local or distant metastases (10). Specifically, the clinical course of SC has been reported to have a moderate risk of local recurrence (15%) and lymph node metastasis (20%) and a low risk of distant metastasis (5%) (1, 11). The present case is considered a typical course of SC because of the local recurrence 20 years after surgery and subsequent slow enlargement.

The pathological diagnosis of SC is usually made using Hematoxylin & Eosin staining and immunostaining (S-100, mammaglobin, and Pan-Trk). Furthermore, to identify cases where *NTRK* mutation is not evident, the presence of the *ETV6-NTRK3* fusion gene is required, which can be confirmed through FISH or reverse transcription polymerase chain reaction (1, 6, 7). In particular, the usefulness of MUC4 for SC and Pan-Trk for *ETV6-NTRK3* fusion in immunostaining has recently been reported. *NTRK* fusion genes are expressed in 0.3–1.6% of all solid tumors, but the expression rate varies widely among cancer types (12, 13). Among them, salivary gland SCs are known to have a high rate (80–100%) of NTRK fusion gene expression, most of which are *ETV6-NTRK3* fusion genes (7, 14).

Recently, the rearrangement of the *ETV6-NTRK3* gene was identified as a therapeutic target (15, 16). The safety and antitumor activity of entrectinib, a potent oral inhibitor of the tyrosine kinases TRKA/B/C, ROS1, and ALK, have been demonstrated in several clinical trials in patients with advanced or metastatic solid tumors harboring genetic rearrangements of *NTRK1-3*, *ROS1*, or *ALK*, regardless of the histological type (17, 18). In an integrated analysis of phase 1-2 trials (STARTRK-1, STARTRK-2, and ALKA-372-001) of solid tumors with the *NTRK* fusion gene, the response rate to the TRK inhibitor entrectinib was 57%, and the median progression-free survival was 11.2 months (5). Another TRK inhibitor, larotrectinib, is also effective in the treatment of solid tumors with the *NTRK* fusion gene (19) and has been covered by insurance in Japan since March 2021, following insurance coverage of entrectinib. Adverse events of entrectinib in this patient included dysgeusia, fatigue, dizziness, weight gain, and creatinine increase, all of which were reported in the integrated analysis of phase 1-2 trial (5), and a reduction in the dose of entrectinib reduced these symptoms. This patient has been continuing entrectinib therapy 30 months after the start of treatment and has been living comfortably.

Thus, recognition of SC and testing for *ETV6-NTRK3* gene rearrangements are critical for patient care. Although there has already been a case report on the sustained efficacy of entrectinib in SC with the *ETV6-NTRK3* gene fusion (20), the number remains small. This case showed a typical clinical presentation of SC in the parotid gland, and complete response was maintained after 30 months of entrectinib treatment. However, without insurance coverage for CGP testing, the patient would not have benefited from entrectinib. Ultimately, our plan is to continue administering entrectinib as long as the therapeutic effect is sustained and side effects remain tolerable.

## Data availability statement

The datasets presented in this article are not readily available because of ethical/privacy restrictions. Requests to access the datasets should be directed to the corresponding author.

## Ethics statement

Written informed consent was obtained from the participant/patient(s) for the publication of this case report.

## Author contributions

EM and KM provided patient care and wrote the manuscript. TO and HU were responsible for patient surgery and genetic diagnosis. JA and AK were responsible for pathology. SN, TT, and YS participated in data acquisition and critical revisions. FF and TK supervised this case report. All authors discussed the results and contributed to the final manuscript.
