# Supplementary material for: Case Report: A case of complete response to entrectinib in NTRK fusion gene-positive parotid gland cancer
Source: Front Oncol. 2023 Aug 4;13:1247435. doi: 10.3389/fonc.2023.1247435 (PMC10436465; doi:10.3389/fonc.2023.1247435)
Supplement: Supplementary file 1 [file Table_1.docx]

**Table**

Supplementary Table 1. Pre-treatment laboratory data

| **Biochemistry** |  | **Peripheral blood** |  |
| --- | --- | --- | --- |
| Total protein | 7.8 g/dL | White blood cell | 6,900 /μL |
| Albumin | 3.7 g/dL | Neutrophil | 58.8% |
| Total bilirubin | 0.9 mg/dL | Lymphocyte | 28.4% |
| Aspartate aminotransferase | 37 IU/L | Monocyte | 8.8% |
| Alanine aminotransferase | 32 IU/L | Eosinophil | 3.3% |
| Lactate dehydrogenase | 240 IU/L | Basophil | 0.7% |
| Alkaline phosphatase | 89 IU/L | Red blood cell | 368×10^4^ /μL |
| γ-glutamyl transpeptidase | 29 IU/L | Hemoglobin | 13.7 g/dL |
| Blood urea nitrogen | 9 mg/dL | Hematocrit | 39.1% |
| Creatinine | 0.67 mg/dL | Platelet count | 20.9×10^4^ /μL |
| Uric acid | 8.9 mg/L |  |  |
| Na | 142 mEq/L |  |  |
| K | 4.1 mEq/L |  |  |
| Cl | 106 mEq/L |  |  |
| Ca | 9.3 mg/dL |  |  |
| C-reactive protein | 0.15 mg/dL |  |  |
